# Vaccination with recombinant oncosphere antigens reduces the susceptibility of sheep to infection with *Taenia multiceps*^☆^

**DOI:** 10.1016/j.ijpara.2007.11.006

**Published:** 2008-07

**Authors:** Charles Gauci, Gulay Vural, Taraneh Öncel, Antonio Varcasia, Veronica Damian, Craig T. Kyngdon, Philip S. Craig, Garry A. Anderson, Marshall W. Lightowlers

**Affiliations:** a The University of Melbourne, Department of Veterinary Science, Werribee, Vic. 3030, Australia; b Veteriner Kontrol ve Arastirma Enstitusu, Parazitoloji Bolumü, 34890 Pendik, Istanbul, Turkey; c Dipartimento di Biologia Animale, Sezione di Parassitologia e Malattie Parassitare, degli Studi di Sassari, Sassari, Italy; d Cestode Zoonoses Research Group, School of Environment and Life Sciences, University of Salford, Salford, UK

**Keywords:** *Taenia multiceps*, Sheep vaccine, Oncosphere, Antigens, Vaccination, Recombinant

## Abstract

*Taenia multiceps* is a cestode parasite, the larval stage of which encysts in the brain of sheep, goats and cattle causing an often fatal condition. The parasite also causes zoonotic infections in humans. Homologues of the recombinant oncosphere vaccine antigens from *Taenia ovis* and other *Taenia* species were identified in *T. multiceps*. Sequencing of the associated *T. multiceps* genes and cloning of the encoding mRNA has revealed conserved features in the genes and proteins. The *T. multiceps* oncosphere proteins, designated Tm16 and Tm18, contain a predicted secretory signal and fibronectin type III domain. The recombinant Tm16 and Tm18 proteins were successfully expressed in *Escherichia coli* as fusion proteins with GST. The antigens, formulated with Quil A adjuvant, were tested in a vaccine trial in sheep. The antigens stimulated immunity in sheep against challenge infection with *T. multiceps* eggs. Five of nine control sheep died due to a challenge infection with *T. multiceps* whereas none of 20 vaccinated animals died as a result of the parasite challenge (*P* = 0.001). In addition, vaccination with the Tm16 protein, or Tm16 plus Tm18, induced significant protection against the number of parasites encysting in the brain as a result of the challenge infection (*P* = 0.023, *P* = 0.015, respectively). No clear relationship was apparent between the level of specific serum antibody in vaccinated animals and either the presence or absence of parasites or the number of parasites that occurred in some of the vaccinated animals. We believe this study is the first description of recombinant vaccine-related investigations for *T. multiceps*. The recombinant oncosphere antigens identified may allow development of effective vaccination strategies against *T. multiceps* infection in sheep. They raise the potential for the development of a combined vaccine with the *Echinococcus granulosus* EG95 antigen for prevention of *T. multiceps* as well as preventing the transmission of cystic hydatid disease.

## 1 Introduction

*Taenia multiceps* is a cestode parasite, the larval stage of which encysts in the CNS, mainly in the brain. The coenurus larva grows to the size of a hen’s egg and causes neurological symptoms known as gid in infected sheep and goats (Edwards and Herbert, 1982). The infection is commonly lethal and the parasite is a significant cause of economic loss in many parts of the world involved in sheep/goat grazing. The parasite also causes zoonotic infections in humans (Pau et al., 1990; Sabattani et al., 2004), although the condition is believed to be relatively rare.

The definitive hosts of *T. multiceps* are dogs and other canids. Sheep become infected by ingestion of parasite eggs passed in the faeces of the definitive host. The oncosphere larval stage is liberated in the small intestine, penetrates the intestinal mucosa and migrates via the blood to the brain where it encysts and develops over a number of months into a mature, infective coenurus. The lifecycle is completed after scoleces contained within the coenurus are ingested by the definitive host.

The global distribution of *T. multiceps* coincides in many instances with the distribution of hydatid disease caused by *Echinococcus granulosus* (Scala and Varcasia, 2006; Sharma and Chauhan, 2006). A highly effective vaccine has been developed which can reduce hydatid infection in livestock animals (Lightowlers et al., 1996a) and it is believed that widespread use of the vaccine could reduce the transmission of hydatid infection to humans. However, there is little direct economic incentive for livestock owners to utilise the vaccine. Development of a combined gid/hydatid vaccine is one potential strategy which could improve application of the hydatid vaccine by providing an indirect economic incentive to livestock owners to vaccinate against hydatids. In addition, an effective vaccine against *T. multiceps* would be economically valuable in its own right.

Recombinant antigen vaccines have been developed against infection with a number of *Taenia* species including *Taenia ovis* in sheep (Johnson et al., 1989; Harrison et al., 1996), *Taenia saginata* in cattle (Lightowlers et al., 1996b) and *Taenia solium* in pigs (Flisser et al., 2004; Gonzalez et al., 2005). These vaccines utilise one or more of three groups of oncosphere proteins that are known as the 16k, 18k and 45W groups, after their original description in *T. ovis*. As an initial step towards the development of a combined gid/hydatid vaccine, we have investigated the presence of genes related to the 16k and 18k antigen families in the genome of *T. multiceps*, cloned and expressed the genes and undertaken a vaccine trial in sheep with the recombinant proteins against a challenge infection with *T. multiceps*.

## 2 Materials and methods

### 2.1 Parasites and parasite infections

*Taenia multiceps* coenuri were obtained from the brains of naturally infected sheep from the region of Nigde, Turkey. Three, 3 month old dogs of mixed breed were treated with 0.1 ml/kg praziquantel (Droncit, Bayer) to ensure the absence of any cestode infection. Five days later the dogs were infected orally with 40 or 20 *T. multiceps* scoleces. Approximately 8 weeks after infection the dogs were found to have *Taenia* eggs in the faeces. *Taenia multiceps* tapeworms were collected after humane euthanasia using an overdose of suxamethonium chloride. The worms were washed in water and the gravid segments collected and stored at 4 °C in antibiotic water (100 IU penicillin, 0.1 mg/ml streptomycin, 0.25 mg/ml amphotericin B; Sigma) and used for DNA/RNA extraction. Subsequently, a second *T. multiceps* infection was undertaken in two dogs and mature eggs were obtained from fresh gravid tapeworm segments obtained from the dogs’ faeces. Eggs were released from the segments using scissors and counted using a Neubauer counting chamber prior to challenge infection of sheep. All animal experiments received ethics approval from the Animal Ethics Committee of the Veteriner Kontrol ve Arastirma Enstitusu, Pendik, Istanbul, Turkey.

A specimen of *T. multiceps* genomic DNA was also obtained from a coenurus isolated from a naturally infected sheep from Wales, UK.

### 2.2 Isolation of parasite nucleic acids

Tapeworm specimens were obtained and genomic DNA was extracted as described previously (McManus et al., 1985; Waterkeyn et al., 1995). Total cellular RNA was extracted from *T. multiceps* eggs according to Puissant and Houdebine (1990) using TRIZOL (Invitrogen).

### 2.3 PCR amplification and cloning of tm16 and tm18 genes

PCR was used to amplify the *tm*18 gene from *T. multiceps* genomic DNA (Welsh parasite sample) using *Pfx* DNA polymerase (Invitrogen) as detailed in Gauci et al. (2006) using the following primers: 5′-GACGTTCACGACGACGAAGATG-3′; 5′-CATTACTAACACCCTGTATTTGTAATCG-3′. Duplicate PCR products were separated by agarose gel electrophoresis, purified using a QIAquick Gel Extraction Kit (Qiagen), A-tailed using Taq DNA polymerase (Promega) according to the manufacturer’s instructions and cloned into a pGEM-T Easy vector (Promega). The *tm*16 gene was amplified and cloned, as above, using the following PCR primers: 5′-CCGGAATTCATGCTGCTTCAAGTTTGCCTC-3′; 5′-CCGCTCGAGGCCAACTGAGACAACACCTG-3′. Primers were designed to amplify the full gene sequence (2594 bp for *tm*16 and 1395 bp for *tm*18).

### 2.4 RT-PCR amplification of tm16 and tm18 mRNA

Total RNA from *T. multiceps* eggs (Turkish parasite sample) was reverse transcribed using Superscript II Reverse Transcriptase (Invitrogen) using oligo(dT) priming as detailed in Gauci et al. (2006). The egg cDNA was used as a template for PCR amplification using *Pfx* DNA polymerase (Invitrogen). For amplification of the *tm*16 cDNA, the following primers were used: 5′-CCGGAATTCGAGGGATACGTTGAATTTGGCG-3′; 5′-CCGCTCGAGGCCAACTGAGACAACACCTG-3′. The following primers were used for amplification of the *tm*18 cDNA: 5′-CCGGAATTCGGTGGCCAAGATTCCAACAAACG-3′; 5′-CCGCTCGAGCATTACTAACACCCTGTATTTGTAATCG-3′.

### 2.5 Cloning into the pGEM-T vector

Duplicate PCR-amplified products were separated by electrophoresis through Tris–Acetate–EDTA (TAE) agarose, excised from the gel and purified using a QIAquick Gel Extraction Kit (Qiagen). Separate product bands were cloned into the plasmid vector pGEM-T using the pGEM-T Vector System I cloning kit (Promega). *Escherichia coli* JM109 was used as the host cell line for the recombinant plasmids.

### 2.6 DNA sequence analysis

The Wizard Plus DNA Purification kit (Promega) was used to prepare recombinant plasmids for use as template in sequencing reactions. Sequencing was performed using the Applied Biosystems PRISM™ automated sequencing system with the ABI BigDye™ Terminator sequencing kit. DNA and protein sequence was compiled using Accelrys DS Gene 2.5. PCR amplification reactions were performed in duplicate and products were cloned/sequenced separately to ensure sequence accuracy.

### 2.7 Bacterial expression and preparation of recombinant antigens for vaccination

The *tm*16 and *tm*18 cDNAs were excised from pGEM-T by double digestion with EcoRI and XhoI, separated from the plasmid by agarose gel electrophoresis and purified from the gel using a QIAquick Gel Extraction Kit (Qiagen). The recovered cDNA inserts were ligated to EcoRI and XhoI digested pGEX-1TEX (GE Healthcare Biosciences) and transformed into *E. coli* by electroporation. The primers used to amplify *tm*16 and *tm*18 from egg mRNA, for subsequent *E. coli* expression, were designed to produce recombinant proteins that were truncated by removal of the predicted secretory signal sequence of each antigen. This resulted in deletion of the 16 N-terminal amino acids of Tm16 and 18 N-terminal amino acids of Tm18. *Escherichia coli* BL21 DE3 was used for expression of the recombinant antigens as fusion proteins with GST (Smith and Johnson, 1988). Super optimal broth (SOB) and 100 μg/ml ampicillin were inoculated with a bacterial colony from an SOB agar culture and incubated in a shaker incubator (Infors, Switzerland) at 37 °C, 210 rpm for 16 h. The overnight culture was diluted 1:10 in SOB containing ampicillin and incubated further with shaking until the O.D._595nm_ reached approximately 0.5–1 U. Recombinant protein expression was induced by addition of isopropyl β-d-thiogalactopyranoside (IPTG) at a concentration of 0.2 mM with incubation for a further 5 h. Bacteria were recovered from the culture medium by centrifugation at 5000*g* and lysed by sonication using a Vibra Cell VCX750 Sonicator (Sonics, CT, USA) fitted with a 19 mm probe. The lysates were clarified by centrifugation at 40,000*g* at 4 °C for 15 min. The GST fusion proteins were purified by batch affinity chromatography according to Smith and Johnson (1988). The purity of the GST fusions was determined by SDS–PAGE and scanning densitometry (Molecular Dynamics). Protein concentration was estimated using the method of Bradford (1976). The purified GST fusion proteins were mixed with Quil A (Superfos, Denmark) and sixfold w/w maltose followed by lyophilisation for transport and storage. The Tm16 and Tm18 antigens were reconstituted in sterile distilled water prior to immunisation of lambs.

### 2.8 Bacterial expression and preparation of recombinant antigens for ELISA

The *tm*16 and *tm*18 cDNAs were excised from pGEX-1TEX by double digestion with EcoRI and XhoI, and cloned into EcoRI/SalI digested pMAL-C2 (New England Biolabs). The *E. coli* JM109 strain was used for expression of the recombinant antigens as fusion proteins with maltose-binding protein (MBP). Culturing and recovery of MBP fusion proteins was performed as described in Section 2.7 and Maina et al. (1988), except affinity purification was performed using amylose resin (New England Biolabs) and fusion proteins were eluted by the addition of 10 mM maltose.

### 2.9 Vaccination trial with recombinant antigens

Thirty sheep, Akkaraman breed, aged 3–4 months were divided into five groups. Three groups of seven sheep each were immunised on three occasions, 2 weeks apart, with affinity purified fusion protein. One group was immunised with 50 μg Tm16, one group with 50 μg Tm18 and one group with 50 μg of each Tm16 and Tm18. One group of five sheep was immunised with 50 μg GST and the remaining four sheep received no immunisations. All immunisations were given by s.c. injection in the neck in a volume of 2 ml together with 1 mg Quil A adjuvant (Superfos, Denmark). Two weeks after the third injection, all sheep were each infected with 5500 *T. multiceps* eggs by intra-rumen injection through a 10 cm, 16 gauge needle. Four months after infection, the sheep were humanely slaughtered and the brain examined for *T. multiceps* coenuri. The number and size of cysts, as well as their location, were recorded using the scheme previously described by Edwards and Herbert (1982). During the experiment blood samples were obtained at 2 week intervals via the jugular vein.

### 2.10 ELISA

Lambs vaccinated with Tm16, Tm18 or both proteins (as GST fusion proteins) were assessed for specific antibody against the respective immunising antigens. The recombinant proteins, fused to MBP, were bound to 96-well microtitre plates (Greiner Bio-One) by incubating 0.25 μg per well in 50 μl carbonate buffer (10 mM Na_2_CO_3_, 30 mM NaHCO_3_, pH 9.6) for 16 h at 4 °C. The plates were blocked (1 h at 37 °C) with 100 μl PBS (pH 7.2) containing 1% w/v sodium caseinate. After six washes with PBS containing 0.05% Tween 20 (PBST), 50 μl of serial dilutions of sheep serum samples were added to the wells for 1 h at 37 °C. Sheep antisera were diluted in 1% sodium caseinate-PBST. After incubation, the plates were again washed six times in PBST, and 50 μl per well of diluted donkey anti-ovine IgG-horseradish peroxidase (HRP) conjugate (1/5000 for Tm16 and 1/4000 for Tm18, Bethyl Laboratories Inc.) was added in sodium caseinate-PBST, and the plates incubated for a further 1 h at 37 °C. The plates were washed six times with PBST and TMB substrate (45 mM dibasic sodium phosphate, 0.22 mM citric acid, 0.42 mM 3,3′,5,5′-tetramethylbenzidine and 2 × 10^−2^% H_2_O_2_) was added (100 μl/well). The reaction was stopped after 30 min by the addition of 2 M H_2_SO_4_ (50 μl/well). The O.D. was measured at 450 nm (Dynex Technologies Ltd., UK). Antibody titres were calculated for serum samples as the dilution of sera that approximately equaled an O.D. of 1.0 and are expressed as the reciprocal of that dilution. Positive and negative control sera were tested on each plate and any plates on which the titre deviated from the mean positive control titre by more than 20% were re-assayed (Rothel et al., 1996).

IgG_1_ and IgG_2_ antibody isotypes were measured by the addition of 50 μl per well of a pre-determined concentration of anti-isotype monoclonal antibody (Rothel et al., 1996; Woollard et al., 1998) for 1 h at 37 °C. Plates were washed and 50 μl per well of a 1/1000 dilution of sheep anti-mouse IgG-HRP (Sigma) was added for a 1 h incubation at 37 °C. Plates were washed and developed as described above.

### 2.11 Statistical methods

The Mann–Whitney *U* test was used for comparison of the number of parasite coenuri or antibody titres found in animals in different groups. Fisher’s exact test was used for comparing the proportion of animals which died following the parasite infection. Spearman’s rank correlation coefficient determined the association between the antibody titre and the number of cysts in vaccinated animals. The proportion of cysts in the parieto-occipital location was compared between the vaccinated and control animals using an exact test for correlated binary data (Corcoran et al., 2001), as implemented in StatXact 8.0 (Cytel Inc., Cambridge, MA). Antibody titres are presented as mean ± SD A two-tailed *P* value <0.05 was considered to be statistically significant.

## 3 Results

### 3.1 Gene cloning and structure analysis

**Fig. 1:**
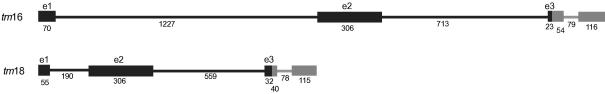
Diagrammatic representation of the gene structure of *tm*16 and *tm*18. Each gene has a conserved structure that consists of three exons (represented by black boxes labelled e1–e3) and two introns (lines) in the protein-encoding region. Grey lines represent introns in the 3′ untranslated region (UTR) and grey bars show 3′ UTR regions that have been identified in the cloned mRNA of each gene. Numbers below each gene represent the length, in bp, of each intron or exon.

**Fig. 2:**
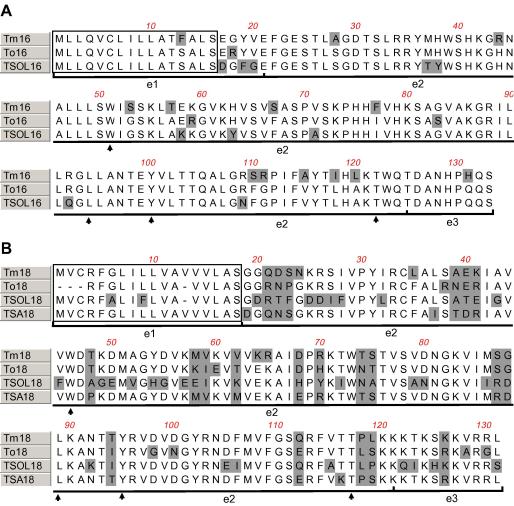
Alignment of the amino acid sequence predicted from cloned mRNA of *tm*16 (A) and *tm*18 (B) from *T. multiceps* with oncosphere antigens from other *Taenia* species. Amino acid mismatches are shaded. Boxed amino acids (1–16 for Tm16, A, and 1–18 for Tm18, B) encode putative secretory signals. Horizontal lines below the alignments define the polypeptide regions encoded by the three exons (e1–e3). Key amino acid residues defining the fibronectin type III domain are highlighted with arrows. GenBank Accession Nos. are as follows; To16 (U89944), TSOL16 (AY147841), To18 (U89943), TSOL18 (AF017788) and TSA18 (X98577).

PCR amplification from *T. multiceps* genomic DNA using primers based on the 16k and 18k genes of *T. ovis* and *T. solium* (Harrison et al., 1996; Gauci et al., 1998; Gauci and Lightowlers, 2003) revealed products which suggested that homologues of these genes occurred in *T. multiceps*. A 2594 bp gene product (*tm*16) and a 1395 bp product (*tm*18) were amplified, cloned and the DNA sequence of each was determined (GenBank Accession Nos. EF672037 and EF672035, respectively). DNA sequencing revealed that both the *tm*16 and *tm*18 genes from *T. multiceps* consist of two exons separated by three introns (Fig. 1). The positions of the protein-encoding regions were confirmed for each gene by amplification and cloning of *tm*16 and *tm*18 transcripts from *T. multiceps* egg mRNA. The cDNA sequence of *tm*16 and *tm*18 confirmed that *tm*16 comprises an open reading frame of 399 bp while *tm*18 has a 393 bp open reading frame. The predicted amino acid sequence deduced from the cloned cDNA of *tm*16 and *tm*18 is shown in Fig. 2. *Tm*16 consists of 133 amino acids and has a predicted mol. wt of 14.79 kDa and an estimated p*I* of 10.05. *Tm*18 consists of 131 amino acids and has a predicted mol. wt of 14.65 kDa and a p*I* of 10.31. Database searches with Tm16 and Tm18 using BLAST (Altschul et al., 1997) identified significant BLAST score hits with oncosphere antigens from other taeniid cestodes (shown in Fig. 2). Predicted secretory signals were identified in Tm16 and Tm18 (Fig. 2) using SignalP (Nielsen et al., 1997) and one putative fibronectin type III domain was also identified in each using SMART (Ponting et al., 1999).

### 3.2 Recombinant protein expression

**Fig. 3:**
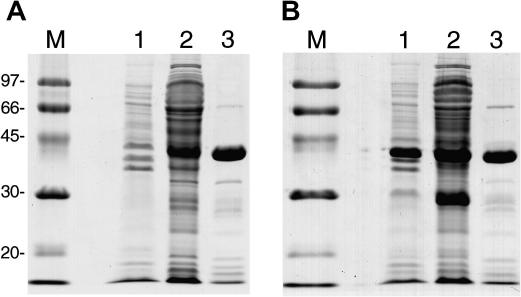
SDS–PAGE of soluble and insoluble protein extracts of *Escherichia coli* cultures expressing GST fusion proteins and affinity purified products. (A) Soluble fraction (lane1), insoluble fraction (lane 2) and purified Tm16-GST (lane 3) from *E. coli* culture expressing Tm16. (B) Soluble fraction (lane1), insoluble fraction (lane 2) and purified Tm18-GST (lane 3) from *E. coli* culture expressing Tm18. Molecular weight markers (kDa) are shown on the left of each panel.

The Tm16 and Tm18 proteins were successfully expressed in *E. coli* as fusion proteins with GST, which facilitated purification of the recombinant proteins from *E. coli* lysates. The cDNA encoding each recombinant protein was genetically modified so that the encoded proteins lacked the amino acid residues that constituted the predicted secretory signals. Tm16 and Tm18 were expressed at moderate levels and were produced as stable, soluble GST fusion proteins (Fig. 3A and B, lane 2) with relative mobilities in SDS–PAGE that correlated with the size of the proteins from the predicted amino acid sequences. The proteins were affinity purified from the soluble fraction of *E. coli* lysate at a purity suitable for application in vaccine trials (Fig. 3A and B, lane 3). The truncated Tm16 and Tm18 proteins were also soluble when expressed as fusion proteins with MBP (data not shown), allowing the use of the MBP fusions in ELISA for the measurement of Tm16- and Tm18-specific immune responses in vaccinated sheep.

### 3.3 Vaccine trial of sheep and experimental challenge with *T. multiceps*

**Fig. 4:**
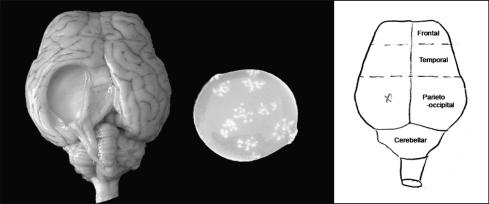
Sheep brain following experimental infection with *Taenia multiceps* and a coenurus removed from the left parieto-occipital region. The scheme used for notation of the location of coenuri in the brains of infected sheep is shown on the right-hand side (after Edwards and Herbert, 1982).

Results of the *T. multiceps* challenge infection in sheep vaccinated with Tm16 and Tm18 are summarised in Tables 1 and 2. Coenuri ranging in sizes up to 45 mm in diameter were found (Fig. 4). All control sheep were found to have *T. multiceps* at autopsy. There was no statistical difference (*P* = 0.30) between the number of cysts found in the non-immunised group (one, three, three, five and twelve parasites) and GST-immunised group (two, two, three and three parasites) and for subsequent analyses, the data from these two control groups were pooled. Five of the nine control sheep died as a result of infection with *T. multiceps*. Four sheep died during the acute phase of the infection and one died during the chronic phase prior to the time at which the remaining sheep were necropsied. One sheep in the Tm16 group died of causes unrelated to the experiment and no infection data were available for this animal. None of the 20 remaining immunised sheep died as a result of the challenge infection (all vaccinated animals versus controls, *P* = 0.001). Immunisation with Tm16 induced statistically significant protection against challenge infection with *T. multiceps* compared with controls (*P* = 0.023; Mann–Whitney test, Table 1). Three out of the six sheep vaccinated with Tm16 harboured no coenuri. Immunisation of sheep with Tm16 and Tm18 combined, also induced significant protection against infection (*P* = 0.015), with four out of the seven animals containing no coenuri. Although four out of the seven sheep vaccinated with Tm18 alone were parasite-free, the number of cysts found in this group was not significantly different compared with control animals (*P* = 0.051). Immunisation with Tm16 and/or Tm18 affected the location of cysts that were found in vaccinated animals compared with cysts in the control animals (Table 2). The vaccinated animals had 7/28 (25%) cysts in the parieto-occipital location, whilst there were 26/34 (76%) in this region in the control animals (*P* = 0.027 after adjusting for multiple cysts per animal). The proportions of cysts in the parieto-occipital location in each of the nine vaccinated sheep were 2/4, 0/1, 0/1, 1/1, 2/7, 0/3, 1/1, 1/1 and 0/9, whilst the proportions in the nine infected control animals were 0/1, 2/2, 3/3, 2/2, 3/3, 1/3, 9/12, 3/5 and 3/3. Due to the death of many control animals during the acute phase of the infection, comparisons were not done for differences in the sizes of cysts in vaccinated and control animals.

### 3.4 Antibody responses

**Fig. 5:**
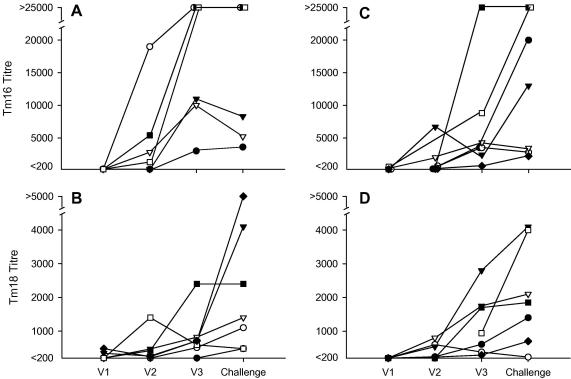
Specific serum antibody responses detected in ELISA to Tm16 and Tm18 antigens in sheep receiving three immunisations (V1–V3) with 50 μg of either antigen alone (A and B) or receiving 50 μg of each protein (C and D) plus 1 mg Quil A adjuvant. All animals were challenged with *T. multiceps* 2 weeks after the third injection. Different symbols represent serum antibody responses in individual animals.

**Fig. 6:**
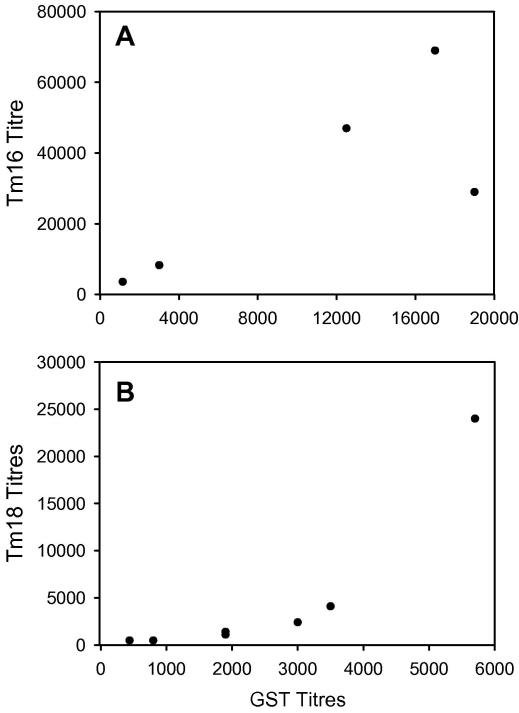
Specific serum antibody responses detected in ELISA to the recombinant antigens (ordinate) and GST (abscissa) in sheep vaccinated with the Tm16 (A) and Tm18 (B) antigens.

**Fig. 7:**
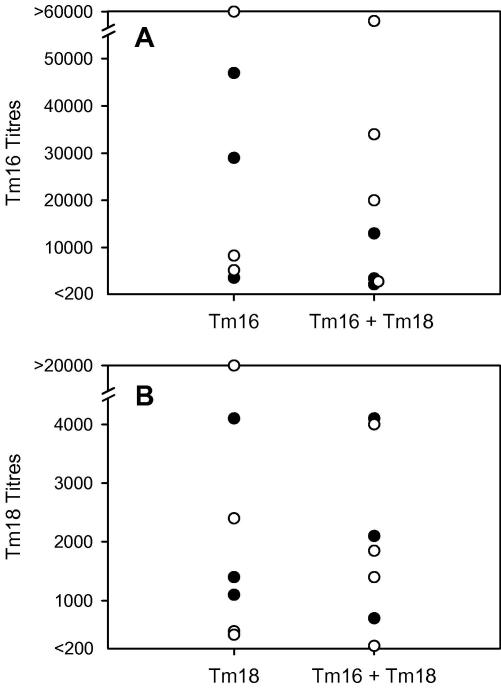
Relationship between the titre of specific antibody against Tm16 or Tm18 antigens detected in individual animals at the time of challenge infection and the presence (solid symbols) or absence (open circles) of parasites detected at necropsy. Groups of sheep were vaccinated either with the Tm16 or Tm18 antigen alone or with a combination of both Tm16 and Tm18. A and B represent total-specific IgG to Tm16 and Tm18, respectively.

Serological analyses of the antibody responses in sheep vaccinated with Tm16, Tm18, or both proteins, indicated that all sheep had seroconverted from being seronegative prior to immunisation to having readily detectable levels of specific IgG antibody against the corresponding protein(s) at the time of challenge infection (Fig. 5). All sera were seronegative for antibodies to MBP (data not shown), allowing the MBP fusion proteins to be used to assess Tm16- and Tm18-specific responses. Control sheep remained seronegative for specific antibodies to Tm16 or Tm18 throughout the experiment (data not shown). All sheep immunised with Tm16 or Tm18 as well as the control group immunised with GST developed readily detectable specific antibody responses to GST (Fig. 6). Responses from individual sheep to the three immunisations with Tm16 or Tm18 varied markedly as did the antibody titre at the time of challenge, although there was a tendency for each immunisation to lead to a boost in specific antibody titre (Fig. 5). Antibody responses at the time of challenge in sheep immunised with Tm16 alone (range 3600–69,000; mean 27,017 ± 26,610) were greater than those raised in sheep vaccinated with Tm18 alone (480–24,000; 4851 ± 8539). With the exception of a single animal which responded to the third immunisation with a prominent rise in titre, sheep vaccinated with Tm18 induced a relatively low titre of specific antibody. This difference in the level of response detected to the Tm16 and Tm18 antigens was also apparent in the animals vaccinated with both proteins (Fig. 5). The level of specific antibody response in Tm18 vaccinated animals was also reflected in relatively low titres of antibody being induced to the GST fusion partner in this group compared with the Tm16 vaccinated group (Fig. 6; anti-GST titres: Tm16 group 10,530 ± 8096, Tm18 group 2463 ± 1795). A clear relationship was not apparent between the titre of specific antibody to Tm16 or Tm18 and the presence or absence of parasites detected at necropsy (*P* = 0.32, *n* = 13 and *P* = 0.56, *n* = 14, respectively; Mann–Whitney) (Fig. 7). There was no significant correlation between the titre to Tm16 or Tm18 and the number of parasites found in vaccinated animals at necropsy (Spearman’s correlation −0.41, *P* = 0.17, *n* = 13 for Tm16 and Spearman’s correlation 0.12, *P* = 0.69, *n* = 14 for Tm18). Three of seven sheep vaccinated with a combined vaccine containing both Tm16 and Tm18, had anti-Tm16 titres greater than 10,000 and were found to be free of parasites after the challenge infection, irrespective of the antibody titre raised against Tm18. Comparisons of vaccine-specific IgG_1_ and IgG_2_ antibody isotype titres found no significant association in relation to the presence or absence of parasites in individual vaccinated animals (Mann–Whitney *U* test; data not shown).

## 4 Discussion

Homologues of the 16k and 18k families of oncosphere antigens, described previously from various *Taenia* species parasites, were found to be present in *T. multiceps* and have been shown here to be capable of being used as vaccines, inducing a significant level of protection against a challenge infection in sheep with *T. multiceps.* Of the nine control sheep which received the challenge infection, five died as a result of the presence of parasites in the brain, whereas none of 20 animals vaccinated with Tm16 and/or Tm18 died following parasite challenge (*P =* 0.001). In addition, sheep vaccinated with Tm16, either as a single antigen or together with Tm18, were found to have significantly reduced numbers of parasites in the brain following the challenge infection (*P* = 0.023, *P* = 0.015, respectively). There was variability in the location and size of *T. multiceps* coenuri between individual sheep, with the majority of cysts being found in either the temporal or parieto-occipital regions of the brain (Table 2 and Fig. 4). This was consistent with the pattern of coenuri distribution observed by Edwards and Herbert (1982) although they found a slightly higher distribution of coenuri in the frontal and cerebellar regions of the brain compared with this study. Vaccination appeared to have an effect on the site in which cysts were located compared with the control animals, with a smaller proportion of cysts detected in the parieto-occipital region of the brain in vaccinated sheep.

The principal or only immune mechanism associated with protective immune responses against taeniid cestode parasites in their intermediate hosts is antibody and complement mediated lysis of the early developing oncosphere (Rickard and Williams, 1982; Lightowlers et al., 2003). Specific antibodies in the sera of animals vaccinated with oncosphere antigens can be shown to be lethal to the early developing oncosphere in a number of *Taenia* species (Kyngdon et al., 2006) but this has not been determined for *T. multiceps*. For these reasons, we investigated whether the protective responses induced by Tm16 or Tm18 are associated with the induction of specific IgG antibodies. The Tm16 and Tm18 recombinant antigens were expressed as MBP fusion proteins allowing the level of Tm16- and Tm18-specific antibodies to be evaluated in the sera of the sheep which were vaccinated with GST fusion proteins. However, no clear relationship was apparent between the level of specific antibody to Tm16 or Tm18 and the presence or absence of parasites found at necropsy. Also, no correlation was evident between the specific antibody titre and the number of parasites found in vaccinated animals at necropsy (*P* = 0.051; Fig. 7). Nevertheless, inspection of the specific antibody titres in the group vaccinated with both Tm16 and Tm18 suggested a possible protective synergy between these two antigens (Fig. 7). Three animals with the highest anti-Tm16 titres were all found to be free of parasites at necropsy, irrespective of the level of their specific titres to Tm18. Synergic, protective immune responses have been described previously between members of the 18k and 45W families of oncosphere antigens from *T. saginata* (Lightowlers et al., 1996b).

The *tm*16 and *tm*18 genes show conserved DNA sequence and structural features that are also present in related genes from the other taeniid species (Waterkeyn et al., 1995; Gauci et al., 1998; Chow et al., 2001; Gauci and Lightowlers, 2003). After cloning and DNA sequencing of the mRNA encoded by the *tm*16 and *tm*18 genes the structure of the genes was confirmed by DNA sequence alignment. The intron/exon structures of *tm*16 and *tm*18 are similar to the homologous genes in *T. ovis* and *T. solium* (Gauci and Lightowlers, 2003). Exons 1 and 2 of *tm*16 and *tm*18 encode modular proteins that contain a predicted secretory signal and a fibronectin type III domain, respectively. This is supported by amino acid sequence alignments (Fig. 2) which show that the *T. multiceps* antigens share amino acid sequences common to *T. ovis* and *T. solium* and that amino acids identified as critical to the fibronectin type III domain (Lightowlers et al., 2000) are also conserved in Tm16 and Tm18. The conformation of cestode oncosphere antigens has been shown to be important for maintenance of host protective epitopes within the antigens (Woollard et al., 2001) and it is possible that the conformation of Tm16 and Tm18, provided by the fibronectin type III domain, may also be important for host protection.

The *Tm16* and *Tm18* genes and their associated mRNAs were cloned from *T. multiceps* specimens originating from different geographical regions (Wales, UK and Turkey, respectively), however no DNA sequence differences were observed in their protein-encoding regions. The genes were also subsequently PCR-amplified and sequenced from Turkish parasite material recovered from sheep used in this study. No DNA sequence differences were observed for *Tm16* and *Tm18* in the different isolates, despite differences being identified in their *cytochrome c-oxidase* genes (data not shown). This suggests that isolates of *T. multiceps* from different geographical locations may show limited or no antigenic variability, in relation to Tm16 and Tm18, despite the known existence of substantial genetic variation between different isolates of this parasite species (Varcasia et al., 2006).

The levels of protection that were achieved here against infection with *T. multiceps* were lower than the high levels of protection that have been demonstrated with other taeniid cestode vaccines (Johnson et al., 1989; Harrison et al., 1996; Lightowlers et al., 1996a,b, 1999; Flisser et al., 2004; Gonzalez et al., 2005). There are several reasons why this may have been the case. The level of antibody response which was achieved here against the Tm16 and, especially, the Tm18 antigens was relatively low compared with the level of response that has been achieved in sheep against other recombinant taeniid cestode antigens (Rothel et al., 1996; Woollard et al., 1998). The Tm18 antigen does not seem to be a poor immunogen per se because sheep subsequently immunised with this antigen in Australia, using an identical vaccination schedule, were found to have antibody titres substantially higher than those found in sera from the sheep trial in Turkey (data not shown). These differences may be associated with other factors such as the different sheep breeds used. Therefore, potential exists for achieving a higher level of protection by optimising the dose, schedule and formulation of the antigens. Another reason for the relative low level of protection achieved against *T. multiceps* may relate to the different biology of *T. multiceps* compared with the other *Taenia* species for which vaccines have been developed, i.e. *T. ovis*, *T. saginata* and *T. solium.* The metacestode of *T. multiceps* encysts in the brain, while the other species infect skeletal muscle. The brain is an immunologically privileged site and it may be that there is little time for the animal’s immune response to kill the invading *T. multiceps* oncosphere compared with the muscle-encysting *Taenia* species. If a lethal impact has not been inflicted on the *T. multiceps* oncosphere prior to its penetration of the brain, it may thereafter be protected from serum antibodies and complement because of its location in the brain.

We believe this study is the first description of vaccine-related investigations on *T. multiceps*. It provides a basis upon which a practical vaccine against *T. multiceps* could be developed for the sheep intermediate host and may also allow the development of a combined vaccine together with the *E. granulosus* vaccine antigen EG95 (Lightowlers et al., 1996a). Incorporation of the hydatid vaccine within an economically valuable *T. multiceps* vaccine could provide the necessary economic incentive needed to achieve uptake of hydatid vaccination and control of this important zoonotic disease.

## Figures and Tables

**Table 1:** Numbers of *Taenia multiceps* coenuri in brains of sheep immunised with Tm16 and Tm18 compared with control animals

Group	Number of coenuri in individual sheep	*P* value^b^	Number of sheep deaths per group
Controls	**1**, 2, **2**, **3**, **3**, 3, 3, **5**, 12^a^	–	5
Tm16	0, 0, 0, 1, 1, 4	0.023	0
Tm18	0, 0, 0, 0, 1, 3, 7	0.051	0
Tm16 + Tm18	0, 0, 0, 0, 1, 1, 9	0.015	0

a Numbers in bold represent individual animals that died as a result of the *T. multiceps* challenge infection.

b The Mann–Whitney *U* test was used for comparison of the number of coenuri in vaccinated sheep compared with controls.

**Table 2:** The location and sizes of *Taenia multiceps* coenuri in the brains of vaccinated and control sheep

	Number of animals	Brain location				
		Frontal	Temporal	Parieto-occipital	Cerebellar	Total
Vaccinated	9	7^a^ (25%)^b^, 5–10^c^	13 (46%), 3–20	7 (25%), 5–45	1 (4%), 3	28
Controls	9	0	8 (24%), 10–25	26 (76%), 9–25	0	34
						
Total	18	7	21	33	1	62

a Number of cysts.

b Percentage of total cysts in that treatment group.

c Range in size of cysts (diameter in mm in situ).
